# Cerebral vasospasm and wernicke encephalopathy secondary to adult cyclic vomiting syndrome: the role of magnesium

**DOI:** 10.1186/s12883-016-0660-x

**Published:** 2016-08-11

**Authors:** Álvaro Sánchez-Larsen, Tomás Segura, Susana García-Muñozguren, Javier Peinado-Ródenas, Joaquín Zamarro, Francisco Hernández-Fernández

**Affiliations:** 1 Department of Neurology, Complejo Hospitalario Universitario de Albacete, Albacete, Spain; 2 Department of Neurology, Grupo Hospitales de Madrid, Madrid, Spain; 3 Department of Radiology, Complejo Hospitalario Universitario de Albacete, Albacete, Spain; 4 Unit of Interventional Neuroradiology, Department of Radiology, Hospital Universitario Virgen de la Arrixaca, Murcia, Spain; 5 Unit of Interventional Neuroradiology, Department of Radiology, Complejo Hospitalario Universitario de Albacete, Albacete, Spain

**Keywords:** Hypomagnesemia, Wernicke encephalopathy, Cerebral vasospasm, Cyclic vomiting syndrome, Case report

## Abstract

**Background:**

Magnesium has a regulatory role in the excitability of cell membranes, and is also a cofactor in the phosphorylation of thiamine. Hypomagnesemia has been associated with coronary vasospasm, but its role in cerebrovascular pathology is controversial, and cerebral vasospasm exclusively attributable to hypomagnesemia has not been reported in humans.

**Case presentation:**

We report the case of a 51-year-old man in whom uncontrollable vomiting, treatment with omeprazole and thiazide, and renal impairment lead to a severe hypomagnesemia (magnesium below the level of detection in blood tests), which secondarily caused Wernicke’s encephalopathy and vasospasm in multiple cerebral arteries (seen with cerebral angiography and CT angiography) that presented with a complete right hemisphere neurological deficit. These disturbances completely resolved when magnesium levels were normalized and subsequent neuroimaging tests confirmed the resolution of angiographic changes.

**Conclusion:**

Our case suggests that hypomagnesemia should be considered in the differential diagnosis of patients with neurological symptoms and predisposing causes.

**Electronic supplementary material:**

The online version of this article (doi:10.1186/s12883-016-0660-x) contains supplementary material, which is available to authorized users.

## Background

Magnesium is the most abundant intracellular divalent cation and the fourth most abundant element in the body. The majority of this mineral is localized in the bones and soft tissues, only 1 % resides in the extracellular fluids [1]. Magnesium balance is maintained by means of intestinal absorption in the small bowel, mostly in the ileum and distal part of jejunum, and renal excretion. The kidneys are crucial in magnesium homeostasis. 70 % of the magnesium filtered is reabsorbed in the thick ascending limb of the loop of Henle. It is estimated that an adult needs an intake of 255–350 mg daily [1]. Conditions that can lead to hypomagnesemia include alcoholism, diabetes, malabsorption (Crohn’s disease, ulcerative colitis, coeliac disease, short bowel syndrome), gastrointestinal loses (biliary or intestinal fistula, large-volume diarrhea or vomiting) endocrine causes (hyperaldosteronism, hyperparathyroidism, syndrome of inappropriate antidiuretic hormone secretion, hypercalcemia, hypercalciuria), renal disease (renal failure, dialysis, intrinsic tubular disorders) and medication use (diuretics, proton pump inhibitors, antibiotics, chemotherapeutic agents) [1].

The principal function of magnesium in the human body is structural, mainly in bones and teeth. However, according to its ionic characteristics, it also plays a role in the regulation of the excitability of neuromuscular membranes. Magnesium works as a cofactor in many enzymatic reactions such as ATP kinase reactions or the activation of thiamine by its phosphorylation to thiamine pyrophosphate [1, 2]. Magnesium is also an antagonist of the N-methyl-d-aspartate (NMDA) receptor [1, 3], and has direct vasodilatory effects due to various mechanisms. Hypomagnesemia is thought to lead to an increase in vascular smooth muscle tone and reactivity, its association with acute focal vasospasm in coronary arteries has been known for decades [4], albeit cerebral vasospasm exclusively attributable to hypomagnesemia in humans has not been reported. We report a case of vasoconstriction in multiple cerebral arteries and Wernicke’s encephalopathy (WE) secondary to hypomagnesemia.

## Case presentation

**Fig. 1:**
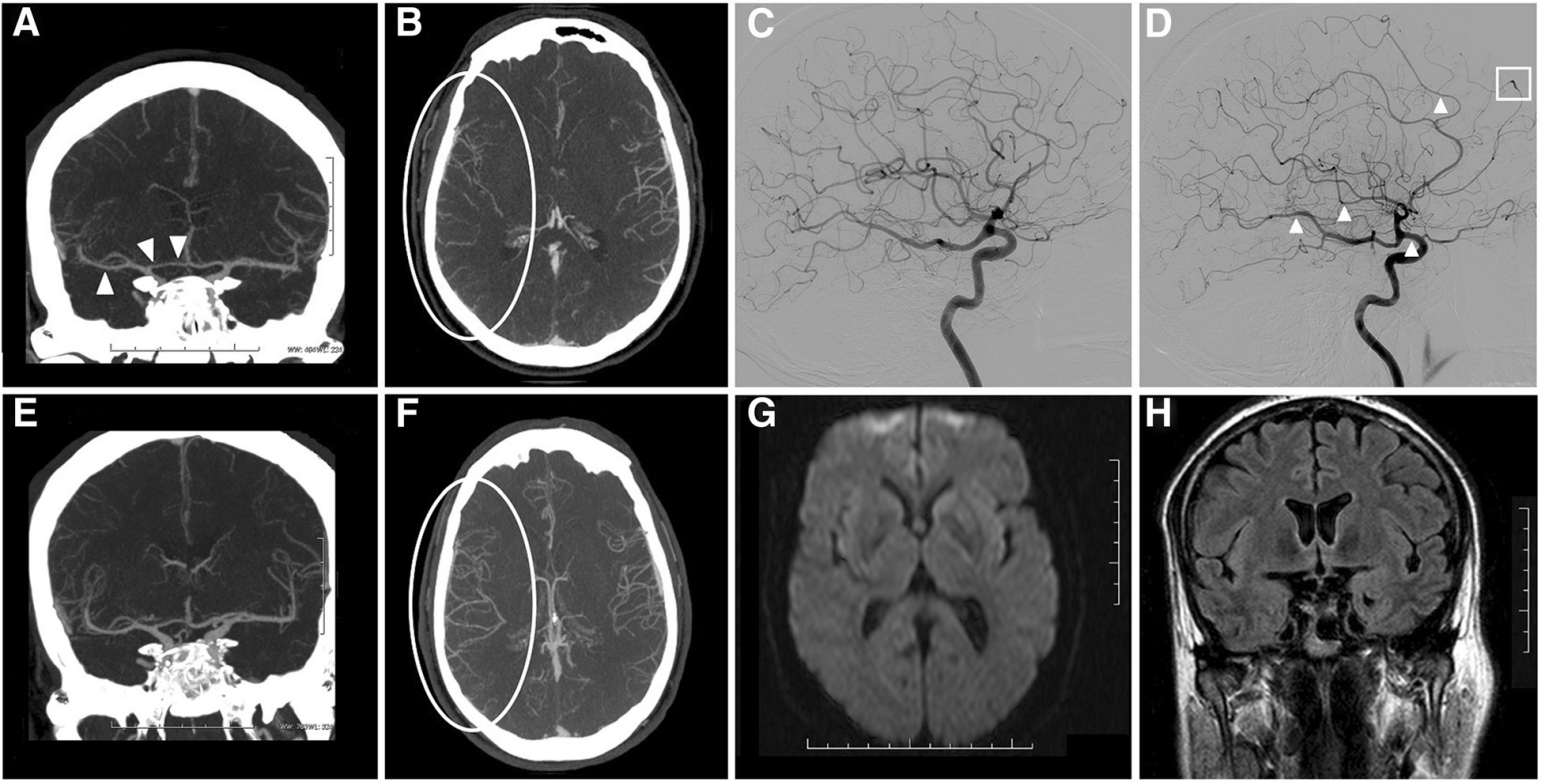
Vasospasm in cerebral arteries secondary to hypomagnesemia. Coronal (**a**) and axial (**b**) CT angiography showing subtle focal changes in the vascular lumen of the right carotid circulation (**a**, white arrows) and overall decrease of the vascularization in the territory of the right middle cerebral artery (**b**, circled in white). Selective cerebral angiography injecting from the left internal carotid artery (**c**), without abnormalities, and from the right internal carotid artery (**d**), showing overall decrease of the vascularization, focal narrowing (**d**, white arrows) and segmental stenosis of several arteries (**d**, white box). CT angiography carried out 7 days after the previous one (**e** and **f**) showing complete resolution of the vascular changes. Diffusion weighted imaging (DWI) (**g**) and fluid attenuation inversion recovery (FLAIR) (**h**) sequences of brain MRI showing no relevant abnormalities

A 51-year-old man, diagnosed with adjustment disorder due to work problems, and hypertension without other vascular risk factors, non-smoker, no alcohol or drug consumption, treated with valsartan/hydrochlorothiazide and omeprazole. He presented with intermittent episodes of incoercible vomiting over a three month period, and was finally diagnosed with adult cyclic vomiting syndrome after an extensive study that ruled out organic origin (normal blood tests, stool test, urea breath test, abdominal CT scan and ultrasound, echoendoscopy with biopsies). The patient was admitted to hospital for prerenal kidney failure after several weeks of uncontrollable vomiting, up to 50 episodes per day. Physical examination was unremarkable except for intermittent optokinetic nystagmus. Blood tests showed creatinine 4.12 mg/dL, urea 92 mg/dL, creatinine clearance 15 mL/min/S, albumin 4.6 g/dL, Na 142 mmol/L, K 3.3 mmol/L, Cl 90 mmol/L, Ca 7.6 mg/dL (normal 8.6–10.2), P 4 mg/dL (normal 2.6–4.5). Intravenous hydration and metoclopramide were started. 24 h later he presented with a sudden and complete right-sided cerebral hemisphere deficit (National Institute of Health Stroke Scale [NIHSS] score of 18), without seizures, fever or other clinical features, and with a blood pressure of 146/93 mmHg. Code Stroke was activated, ECG, brain CT scan and transcranial Doppler were normal and intravenous thrombolysis was decided upon. There was no improvement after 30 min, and a CT angiography was performed (Fig. 1a–b) showing subtle narrowing of the entire right circulation, and stenoses in several branches of the right middle cerebral artery, making it impossible to rule out a distal vessel occlusion. Rescue therapy with mechanical thrombectomy was proposed, but cerebral angiography (Fig. 1c-d) showed several segments with stenosis without occlusion in multiple arteries of the right carotid circulation, so no therapeutic procedure was finally realized. There was no improvement after 24 h, and the patient then developed a rapid progressive encephalopathy accompanied by visual hallucinations, ataxia, multidirectional nystagmus and ophthalmoparesis, symptoms in keeping with WE. Brain MRI was unremarkable (Fig. 1g-h, see also Additional files 1, 2, 3 and 4), with no ischemic areas or other abnormalities. Further complete blood tests were conducted, and magnesium levels were found to be below the level of detection (<0.5 mg/dL, normal 1.5–2.5), mild hypocalcaemia (6.6 mg/dL) and hypophosphatemia (1.9 mg/dL) were also observed. Diuretics, omeprazole and dextrose were stopped, intravenous ondansetron controlled the vomiting, and aggressive intravenous magnesium replacement, saline and empirical thiamine were commenced, and the patient was admitted to the intensive care unit to control potential cardiac arrhythmias. Blood tests after 24 h showed almost normal calcium levels (8.5 mg/dL) and a mild hypomagnesemia persisting (1.3 mg/dL). In subsequent blood tests all ions were always in normal ranges. No cardiac abnormalities were apparent, and following analytical recovery the patient started to improve and within 48 h the focal symptoms disappeared, and WE was completely resolved within two weeks. CT angiography carried out 7 days after the beginning of symptom onset (Fig. 1e–f) showed resolution of the arterial changes. Subsequent studies excluded renal tubular diseases (normal magnesium and other ions in 24–hour urine), endocrine disorders (PTH and 25-hydroxy vitamin D normal) and in successive neuroimaging controls no new abnormalities were found. The patient remains neurologically asymptomatic.

## Conclusions

Acute hypomagnesemia can produce different symptoms depending on the severity of the deficiency. Early signs of hypomagnesemia are non-specific and include loss of appetite, lethargy, nausea, vomiting, fatigue and weakness. The association with hypophosphatemia and hypocalcaemia is frequent. Subsequently, lower magnesium levels can result in tremor, muscle cramps, tetany and generalized seizures [1]. On the other hand, chronic magnesium deficiency is not rare these days, however, it usually remains asymptomatic. This condition has been associated with anxiety, depression, other psychiatric disorders and dementia [1]. Hypomagnesemia is a known cause of Wernicke`s encephalopathy, and frequently these cases are refractory to treatment with thiamine, as magnesium is a cofactor in thiamine phosphorylation, needed for its activation [2]. Our patient developed WE, without an associated Korsakoff syndrome as he did not present with amnesia. These symptoms were completely reversed after two weeks of supplementation with magnesium and thiamine.

As previously mentioned, magnesium plays a key role in the regulation of the excitability of cell membranes. It antagonizes the NMDA receptor [1, 3] and cell surface and intracellular voltage-gated calcium channels [3, 5], thereby impeding calcium entry to ischemic neurons, which is crucial for the activation of cellular apoptotic pathways. Due to these actions, magnesium has been shown to be a neuroprotective agent in different models of cerebral ischemia, demonstrating improvement of prognosis after a cardiac arrest [6], in pre-term infant hypoxic-ischemic injury [7], during cardiac by-pass surgery [8] and during carotid endarterectomy [9]. Nonetheless, recent trials that evaluated intravenous magnesium versus placebo in acute stroke have shown no benefit to this treatment, nor remarkable adverse events [10, 11].

According to its cardiovascular functions, magnesium deficiency has been linked to vascular risk factors such as hypertension, atherosclerosis and diabetes, as well as having a statistical association with greater risk of coronary heart disease, myocardial infarction, sudden cardiac death and stroke [1]. This ion has direct vasodilatory effects in the cerebral vasculature by antagonizing endothelin-1, neuropeptide Y and angiotensin II, whilst at the same time exerting a calcium antagonist effect on vascular smooth muscle [3, 5]. Several trials reported its benefit as a vasodilator agent improving symptomatic vasospasm and delayed ischemia in aneurysmal subarachnoid hemorrhage (SAH) [12, 13], and for years it was used routinely in the treatment of these problems. However, recent trials evaluated the effect of intravenous magnesium for aneurysmal SAH and showed no benefit in any of their objectives [14, 15]. It is possible that prolonged delay of the administration of magnesium and its limited passage through the blood–brain barrier after intravenous supplementation [5, 16] have limited its potential benefit. Furthermore, hypomagnesemia has been previously described as a cause of human coronary [4, 17] and retinal [18] vasospasm, but cerebral vasospasm directly attributable to hypomagnesemia has previously been proven only in animals models [19]. This may be because cerebral circulation is less sensitive to variations in magnesium concentrations due to the blood–brain barrier, and mild or moderate hypomagnesemia may have no effect on the cerebral vasculature. In the case of our patient, massive vomiting, renal impairment and previous treatment with omeprazole and thiazide lead to a severe hypomagnesemia, which caused secondary vasoconstriction in multiple cerebral arteries, in addition to WE. These disturbances were resolved when serum magnesium levels were normalized, which further confirms the cause-and-effect relationship. It is also possible that adult cyclic vomiting syndrome, which has been associated with autonomic vascular changes [20, 21], could have played a role in the cerebral vasospasm of the patient, albeit previous studies did not report any cerebrovascular event, so it is difficult to establish a clear association in this case. As to why our patient developed cerebral vasospasm and not vasospasm at other locations remains unknown. In the same way, we cannot give a feasible explanation for why these changes only occurred in the right hemisphere and not in both. Further studies in this area are needed to confirm these hypotheses.

In conclusion, hypomagnesemia should be considered in the differential diagnosis of patients with neurological symptoms and predisposing causes for magnesium deficit. To the best of our knowledge, we have not found any other reported cases of cerebral vasospasm attributable to hypomagnesemia in humans in the literature. This may be an underdiagnosed condition which should be considered because rapid detection and correction of serum magnesium levels would completely resolve the symptoms. Further studies are needed to confirm these hypotheses.

## Abbreviations

NIHSS, National Institute of Health Stroke Scale; NMDA, N-methyl-d-aspartate; SAH, subarachnoid hemorrhage; WE, Wernicke’s encephalopathy

## Additional files

Additional file 1: MRI, axial T1 sequence of the pons, cerebellum and temporal poles showing no abnormalities. (JPG 265 kb) Additional file 2: MRI, axial T1 sequence of the pons, cerebellum and temporal poles showing no abnormalities. (JPG 417 kb) Additional file 3: MRI, axial T1 sequence of the midbrain, perisylvian spaces and temporo-occipital regions showing no abnormalities. (JPG 395 kb) Additional file 4: MRI, axial T1 sequence of the basal ganglia showing no abnormalities. (JPG 277 kb)
