# Supplementary figures and images for: Cerebral vasospasm and wernicke encephalopathy secondary to adult cyclic vomiting syndrome: the role of magnesium
Source: BMC Neurol. 2016 Aug 11;16:135. doi: 10.1186/s12883-016-0660-x (PMC4982311; doi:10.1186/s12883-016-0660-x)

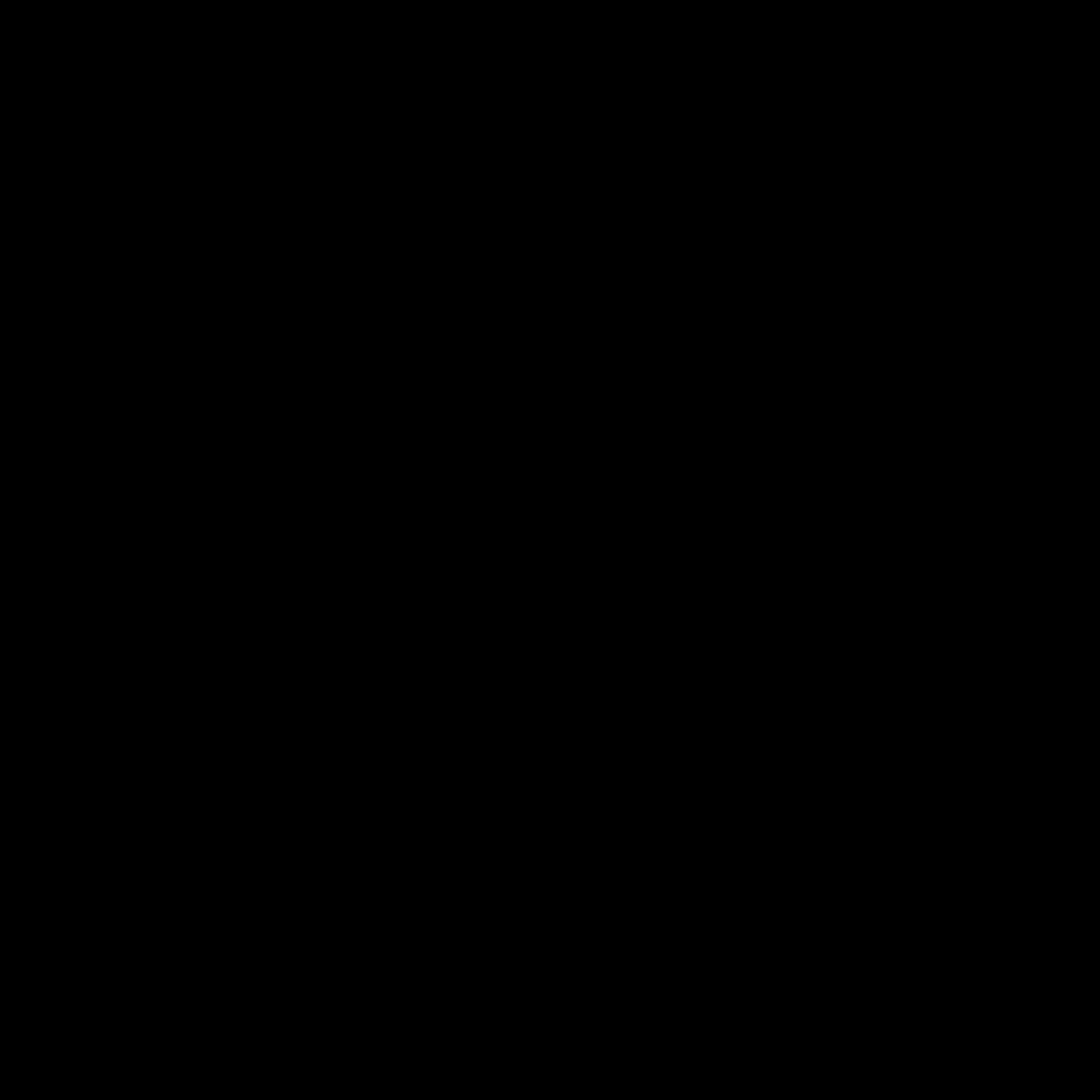

Supplement: Additional file 1: — MRI, axial T1 sequence of the pons, cerebellum and temporal poles showing no abnormalities. (JPG 265 kb) [file 12883_2016_660_MOESM1_ESM.jpg]

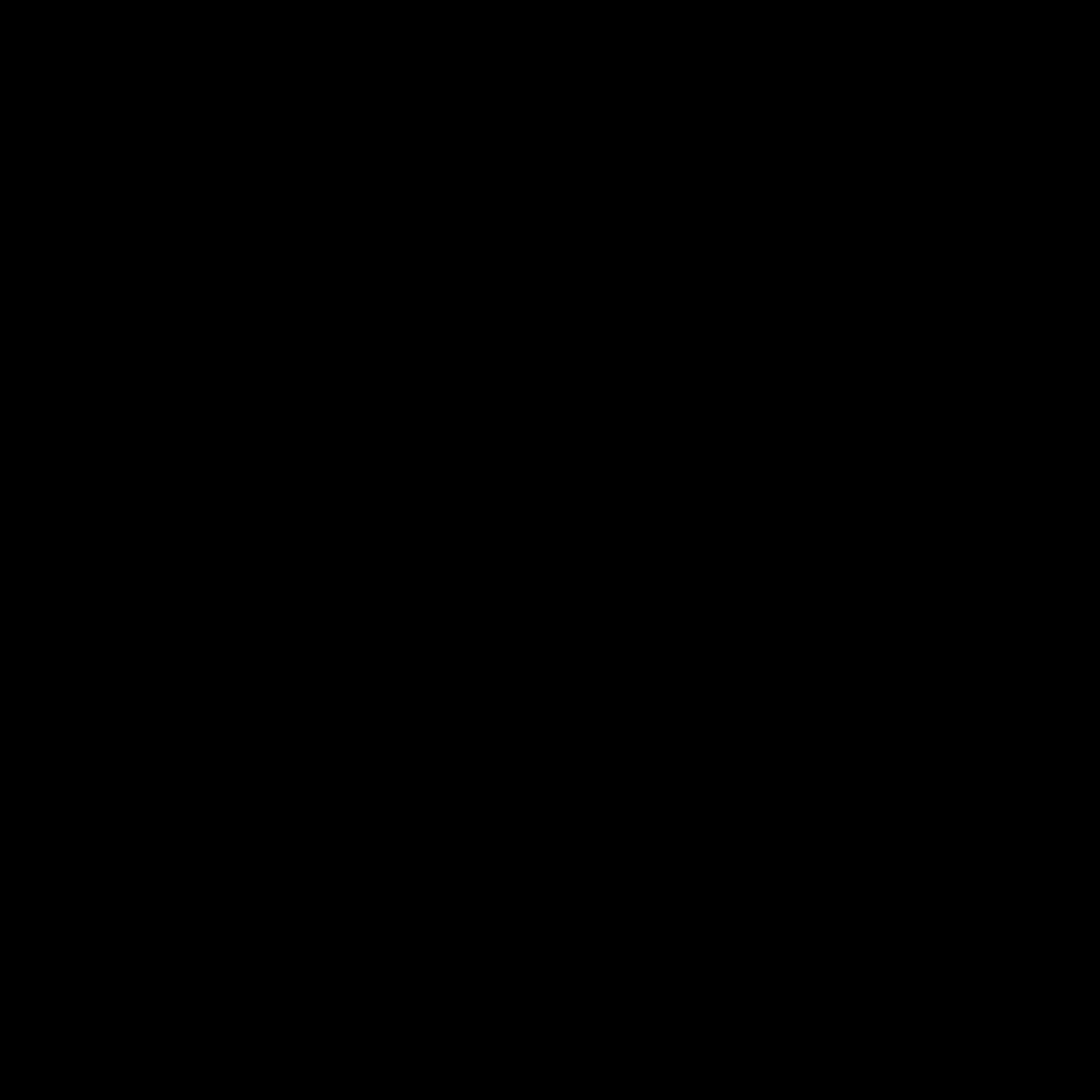

Supplement: Additional file 2: — MRI, axial T1 sequence of the pons, cerebellum and temporal poles showing no abnormalities. (JPG 417 kb) [file 12883_2016_660_MOESM2_ESM.jpg]

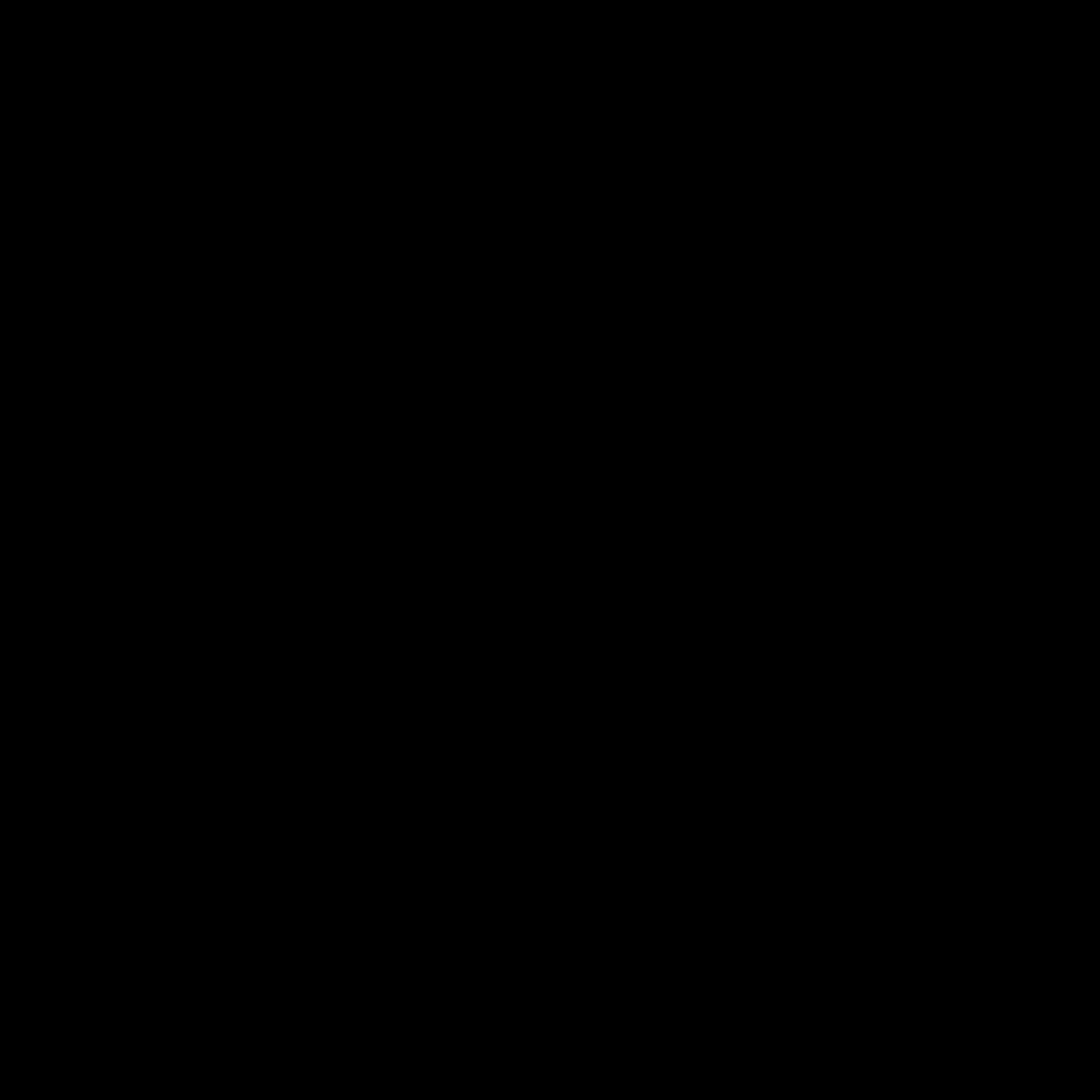

Supplement: Additional file 3: — MRI, axial T1 sequence of the midbrain, perisylvian spaces and temporo-occipital regions showing no abnormalities. (JPG 395 kb) [file 12883_2016_660_MOESM3_ESM.jpg]

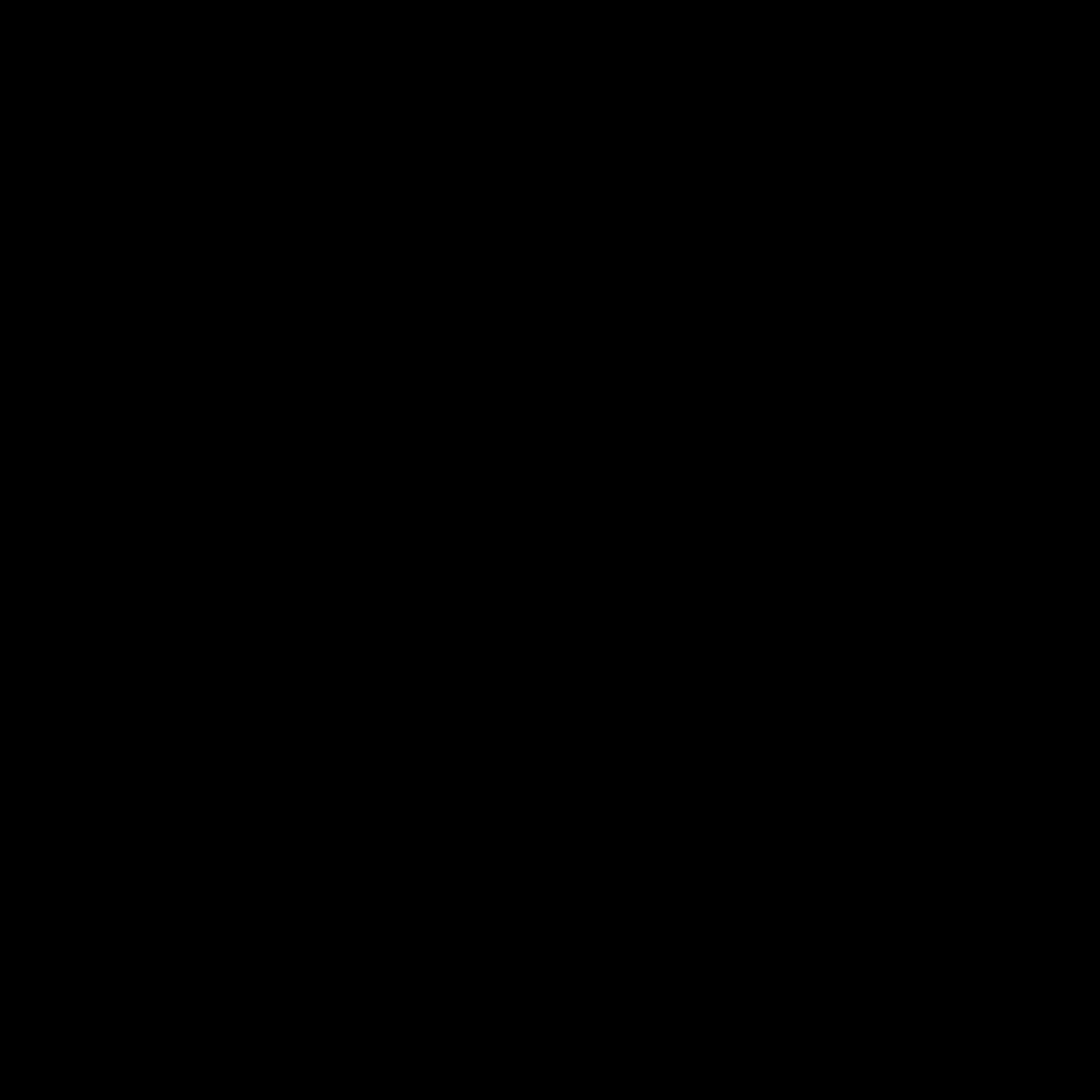

Supplement: Additional file 4: — MRI, axial T1 sequence of the basal ganglia showing no abnormalities. (JPG 277 kb) [file 12883_2016_660_MOESM4_ESM.jpg]
